# Timing-Dependent Neuromuscular Responses to Pulsed Electromagnetic Field (PEMF) Stimulation During Cycling Exercise in Sedentary Males

**DOI:** 10.3390/bioengineering13101165

**Published:** 2026-10-06

**Authors:** Aurelio Trofè, Alessandro Piras, Michela Persiani, Luca Breviglieri, Alessandra Laffi, Andrea Meoni, Milena Raffi

**Affiliations:** 1 Department for Life Quality Studies, University of Bologna, 47921 Rimini, Italy; aurelio.trofe2@unibo.it (A.T.); alessandro.piras3@unibo.it (A.P.); lucabreviglieri@gmail.com (L.B.); 2 Department of Biomedical and Neuromotor Sciences, University of Bologna, 40126 Bologna, Italy; michela.persiani3@unibo.it (M.P.); alessandra.laffi3@unibo.it (A.L.); andrea.meoni@unibo.it (A.M.)

**Keywords:** aerobic exercise, blood lactate, cycling, electromyography, moderate physical activity

## Abstract

Pulsed electromagnetic field (PEMF) increases the amplitude of muscle activity during physical exercise. This study investigated the effects of PEMF stimulation timing on neuromuscular activation in young sedentary males, during moderate-intensity constant-load cycling. We examined whether a PEMF applied at the beginning or during the middle of continuous exercise elicits differential effects on muscle activation. The participants performed a 30 min cycling task at 50% of VO_2_max in which PEMF stimulation was alternately activated or deactivated in 15 min bouts. Surface electromyography (EMG) was recorded from the right vastus medialis and biceps femoris, and blood lactate samples were collected at baseline and during exercise. Data were analyzed via linear mixed-effects modeling. Active PEMF stimulation significantly increased normalized EMG amplitude in the vastus medialis (*p* < 0.001) and biceps femoris (*p* = 0.001) compared to inactive conditions, with the greatest effect observed when stimulation was applied after 15 min of exercise. Blood lactate concentrations were significantly elevated during PEMF application relative to baseline and inactive stimulation (*p* < 0.001). The timing of PEMF stimulation influenced neuromuscular responses during exercise, highlighting its potential as a tool to modulate muscle performance in sedentary individuals.

## 1. Introduction

Physical inactivity represents a major and increasing global health concern. In 2022, approximately 31% of adults worldwide did not meet the recommended levels of physical activity, corresponding to nearly 1.8 billion people, and the global prevalence of insufficient physical activity has increased over the past decade [1,2]. Physical inactivity contributes substantially to the global burden of non-communicable diseases, premature mortality, and health-care costs [3,4,5,6]. Sedentary behavior frequently accompanies low levels of habitual physical activity and has independently been associated with adverse cardiometabolic outcomes and increased mortality [5,6]. Moreover, greater sedentary time has been associated with lower cardiorespiratory fitness and muscular strength in adults [7]. These findings highlight sedentary and physically inactive individuals as a relevant population in which to investigate interventions capable of modulating neuromuscular responses during physical exercise.

Pulsed electromagnetic field (PEMF) represents a non-invasive tool widely used to support physiological function and tissue repair through interactions with biological systems at the cellular level [8,9,10]. PEMF exposure modulates transmembrane potentials and ion fluxes, particularly through the activation of voltage-gated calcium channels, resulting in increased intracellular Ca^2+^ concentrations [10,11]. Elevated Ca^2+^ facilitates Ca^2+^-calmodulin binding and subsequent activation of nitric oxide synthase (NOS), thereby enhancing NO production and downstream vascular and metabolic support. In parallel, PEMF-induced changes in membrane permeability may lower excitation thresholds in motor neurons and muscle fibers, improving neuromuscular excitability and the efficiency of excitation–contraction coupling [12,13]. Consistent with these mechanisms, human studies have reported functional and structural muscle adaptations following PEMF exposure. Clinical trials have documented improvements in mobility and lean body mass when a PEMF was delivered through brief repeated sessions, particularly in older adults and rehabilitation contexts [14]. A PEMF applied after eccentric exercise has been shown to attenuate exercise-induced peripheral nerve dysfunction and promote earlier recovery of force production, suggesting a protective effect on neuromuscular function [15]. In clinical populations, PEMF therapy has also been associated with improvements in pain, joint stiffness, and physical function in patients with knee osteoarthritis [16].

Previous studies in trained participants provided initial evidence that PEMF stimulation can modulate physiological and neuromuscular responses during exercise, including muscle oxygen extraction, muscle activation, and systemic blood lactate concentrations [17,18,19]. However, responses observed in trained individuals cannot necessarily be generalized to sedentary populations. More recently, we showed that PEMF stimulation increased EMG amplitude during moderate-intensity constant-load cycling in young sedentary individuals [20]. This finding provides the direct rationale for the present study, in which we specifically investigated whether the timing of PEMF application further modulates muscle activation in this population. Importantly, increases in EMG amplitude observed during PEMF stimulation reflect genuine physiological modulation. PEMF-related artifacts can be effectively removed through validated signal-processing procedures, allowing isolation of true neuromuscular activity [21,22,23,24] as already reported in a previous study [20]. The consistency of EMG modulation across muscles and stimulation conditions, together with concurrent metabolic responses such as increased blood lactate, further supports a biological origin of the observed effects.

Despite growing evidence supporting PEMF efficacy in musculoskeletal and rehabilitation settings [25,26,27], the biological response to PEMF appears to depend on multiple stimulation parameters, including frequency, intensity, exposure duration, and the characteristics of the target tissue [18]. Recent studies have increasingly investigated PEMF in relation to exercise and skeletal muscle function, using protocols ranging from repeated PEMF combined with resistance or home-based exercise to chronic exposure and post-exercise stimulation [15,26,28,29]. However, considerable variability remains in stimulation protocols, including treatment duration, frequency, and timing relative to exercise, and the optimal parameters have yet to be established [18,19,20,21,22,23,24,25,26,27,28]. In particular, although recent studies have examined PEMF administered in conjunction with exercise or during post-exercise recovery [15,26,28], it remains unclear whether the timing of PEMF application within an ongoing bout of continuous exercise influences the neuromuscular response. Therefore, the aim of the present study was to investigate whether the timing of PEMF stimulation modulates neuromuscular activation during moderate-intensity constant-load exercise in young sedentary individuals. Specifically, we examined whether a PEMF applied at the beginning or during the middle of continuous exercise elicits differential effects on muscle activation. To the best of our knowledge, this is the first study to systematically assess the influence of PEMF timing during ongoing dynamic exercise, providing novel insights into the temporal dynamics of PEMF–muscle interactions.

## 2. Materials and Methods

This study was performed as a single-blind, randomized controlled trial (ClinicalTrials.gov ID: NCT06446466). The study complied with the principles of the Declaration of Helsinki and was approved by the Bioethics Committee of the University of Bologna (protocol code: 23212, approval date: 5 February 2020). All participants were provided with both verbal and written information regarding the study procedures and signed written informed consent prior to enrollment.

### 2.1. Participants

**Figure 1:**
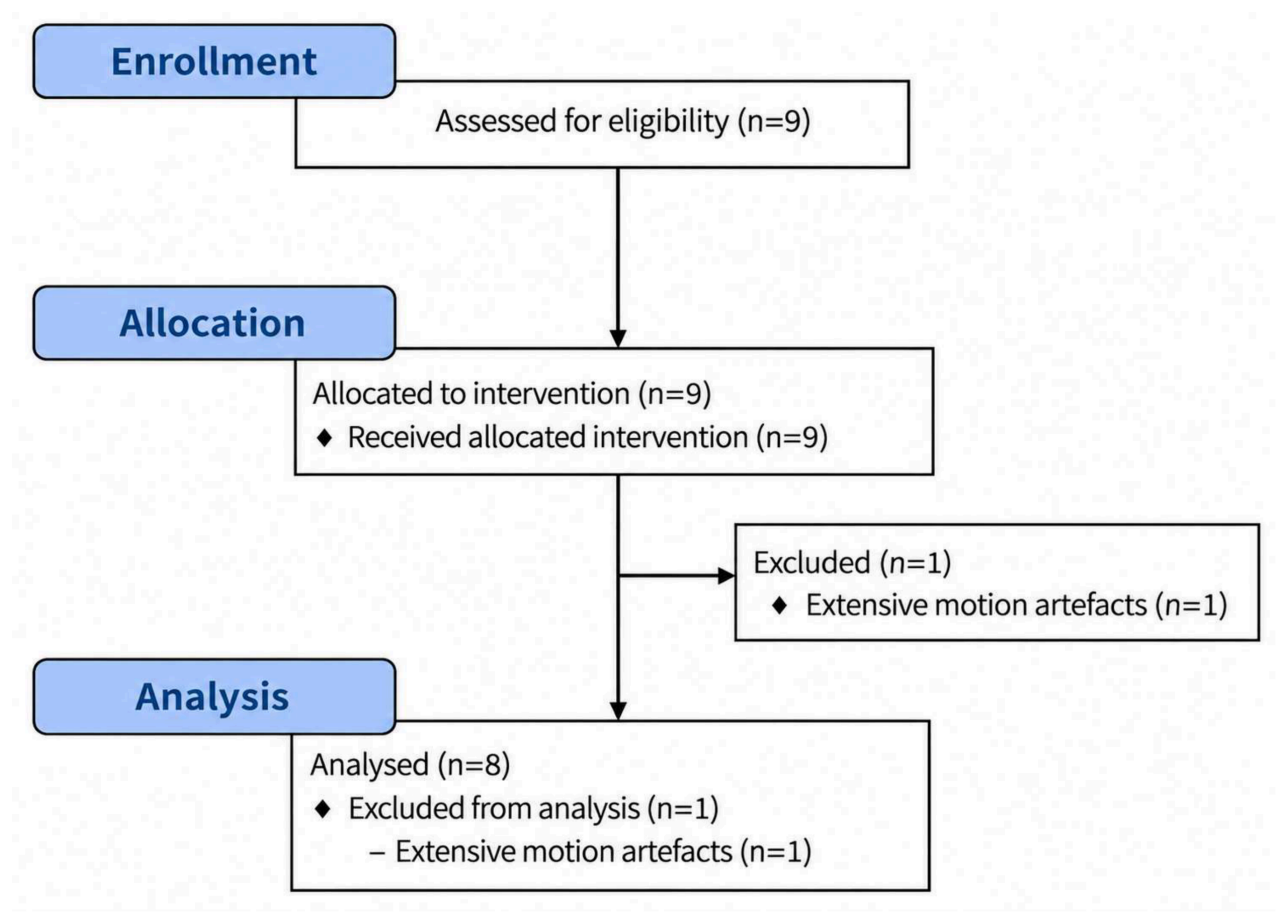
Flow chart of study process.

The dataset of this study is the same presented in a previous paper [20]. Based on an estimated effect size of 0.45 as reported by Trofè et al. [17,19], an a priori power analysis using G*Power software version 3.1.9.2 (Heinrich Heine University Düsseldorf, Düsseldorf, Germany) determined that a sample size of eight participants would provide adequate statistical power (0.80) to detect a significant difference at an alpha level of 0.05. Thus, nine sedentary young adult males were enrolled, with one additional participant recruited to account for potential data loss or corruption. Participant recruitment was restricted to healthy, non-smoking males up to 35 years of age with a sedentary lifestyle, in order to ensure a homogeneous baseline in physical status. The recruitment was restricted to males in order to obtain a physiologically homogeneous sample and to avoid the potential confounding effects of menstrual-cycle-related hormonal fluctuations in this initial pilot study. The anthropometric and physiological characteristics of the participants are shown in Table 1. Exclusion criteria comprised the presence of neuromuscular disorders, physical impairments, psychiatric conditions, and the use of medications or dietary supplements. The study flow chart is shown in Figure 1.

### 2.2. Testing Procedure

Surface EMG activity was recorded from the right vastus medialis (RVM) and the long head of the right biceps femoris (RBF) at a sampling rate of 1000 Hz using a Free-EMG 1000 system (BTS S.p.A., Garbagnate Milanese, Italy). The wireless EMG probes had a 16-bit resolution, an input impedance of 100 MΩ, and a common-mode rejection ratio (CMRR) > 110 dB at 50–60 Hz. EMG signals were acquired at a sampling frequency of 1000 Hz. Electrodes were positioned over the muscle bellies in accordance with SENIAM guidelines (www.seniam.org, accessed on 14 February 2020). Prior to electrode placement, the skin was shaved and cleansed with ethanol to reduce impedance. Bipolar Ag/AgCl disposable electrodes (32 × 32 mm; active area: 0.8 cm^2^; inter-electrode distance: 2 cm; RAM Apparecchi Medicali S.r.l., Genova, Italy) were used.

The Free-EMG system consists of wireless, miniaturized probes designed for dynamic muscle activity analysis. Each probe (41.5 × 24.8 × 14 mm) was directly connected to the electrodes for signal detection and acquisition. Data were transmitted to a PC via USB 2.0 receivers using IEEE 802.15.4 wireless communication. Each probe was equipped with internal memory to ensure continuous data recording in the event of temporary signal loss.

**Figure 2:**
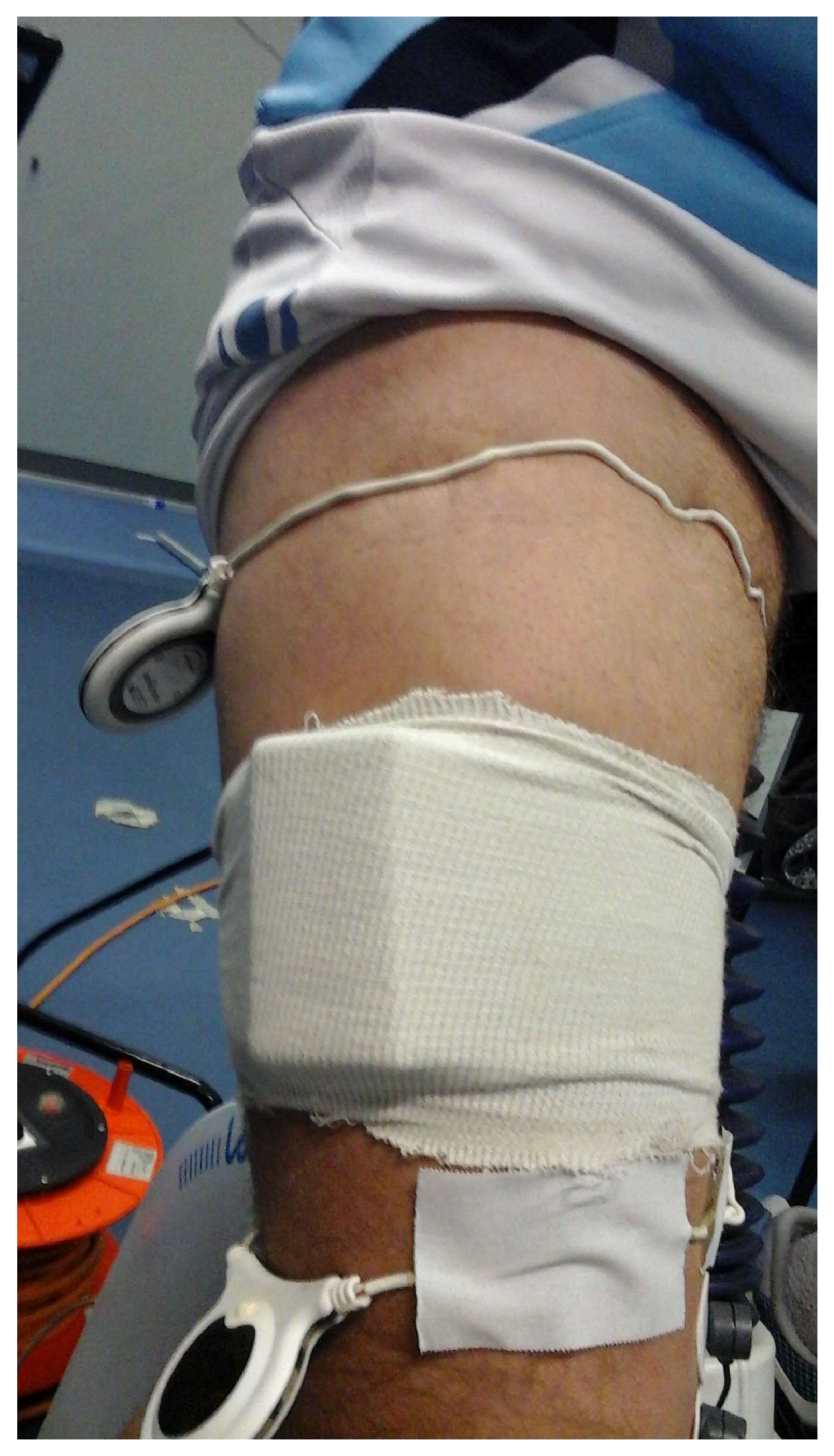
PEMF devices positioned on the belly of the right vastus medialis (RVM) and right biceps femoris caput longum (RBF).

PEMF stimulation was delivered across the right thigh using two circular loop antenna devices (20 cm diameter) from the Torino II^®^ system (Rio Grande Neurosciences, San Francisco, CA, USA), positioned directly over the muscle bellies of the RVM and RBF according to SENIAM guidelines (www.seniam.org), in order to target the whole muscular tight region, as shown in Figure 2. The generated PEMF signal comprised a 27.12 MHz sinusoidal carrier wave modulated into pulse bursts, characterized by a 2 ms burst duration repeating at a frequency of 2 Hz, delivering a peak magnetic field strength of 5 ± 1 µT at the coil’s center.

Experimental sessions were carried out in a temperature-controlled environment (22 °C) and performed on a cycle ergometer (H-300-R; Lode B.V., Groningen, The Netherlands) following a standardized protocol. Subjects were blinded to the stimulation condition and were not informed whether the PEMF was ON or OFF during each trial. The PEMF device was operated by the researcher conducting the experimental session according to the randomized condition assigned to that trial. Data analysis was performed by researchers who were different from those responsible for conducting the laboratory experiments and recording the data. The analysts were aware of the ON/OFF condition. To minimize the influence of circadian variability, all testing sessions occurred between 09:00 am and noon. Participants were instructed to refrain from caffeine, alcohol, and vigorous physical activity for at least 48 h prior to each session.

Each subject attended five laboratory sessions, separated by a minimum interval of three days to ensure adequate recovery.

On Day 1, participants underwent assessments of maximal voluntary contraction (MVC) and maximal oxygen uptake (VO_2_ max). MVC was measured using gym machines (Exere Air Machine, Poggio Renatico, Italy) during isometric contraction, with the leg extension exercise targeting the RVM and the leg curl exercise targeting the RBF. For each muscle, participants performed a sustained isometric contraction against maximal load for five seconds, and the peak MVC value was used to normalize surface EMG data following the same protocol as previously used [30,31]. Subsequently, participants completed an incremental cycling test on the cycle ergometer (H-300-R Lode) to determine individual VO_2_ max with the following protocol: each subject cycled at 50 Watt for 5 min, followed by a workload at 80 watt that increased by 20 Watt every 1 min, at a cadence of 70 RPM, until volitional exhaustion [17]. Respiratory gases were analyzed using a Quark b^2^ breath-by-breath metabolic system (COSMED S.r.l., Rome, Italy). The VO_2_ max values obtained were used to calculate the exercise workload, set at 50% of each participant’s VO_2_ max.

**Figure 3:**
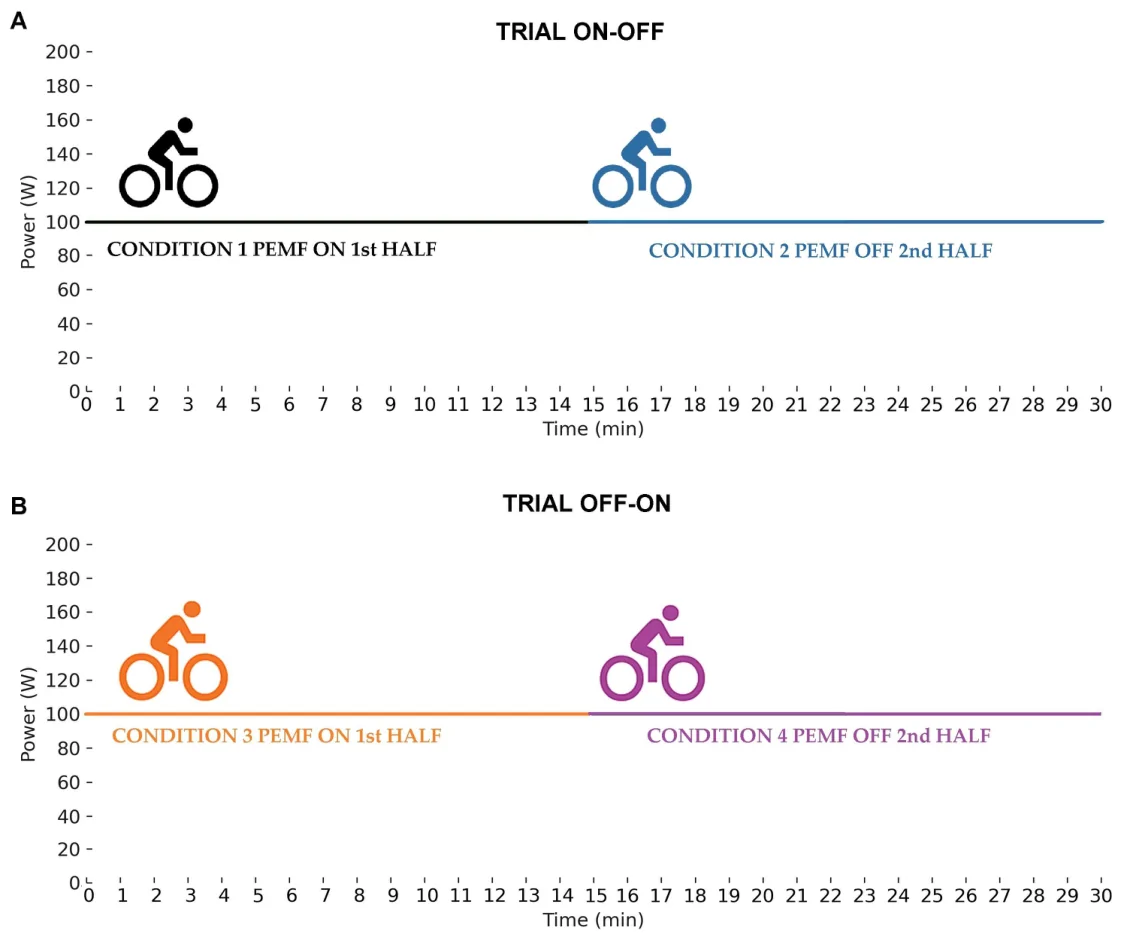
Temporal protocol of the experimental trial. (**A**) ON–OFF trial: The PEMF was activated in the first 15 min (PEMF ON, black) of the exercise and then turned off (PEMF OFF, blue). (**B**) OFF–ON trial: The PEMF was deactivated in the first 15 min (PEMF OFF, orange) of the exercise and turned on in the next 15 min (PEMF ON, purple).

From Day 2 to Day 5, participants completed four experimental exercise sessions, with one 30 min trial performed at each visit. Each participant performed two ON–OFF trials, in which the PEMF was active during the first 15 min and inactive during the second 15 min, and two OFF–ON trials, in which the PEMF was inactive during the first 15 min and active during the second 15 min. Thus, each participant completed a total of four trials. The order of the four trials was randomized independently for each participant before the experimental sessions, so that the two repetitions of the ON–OFF and OFF–ON protocols were distributed in a different sequence across visits. None of the study participants knew whether the PEMFs were turned ON or OFF, either during the exercise or before and after the recording. EMG data were collected continuously during the 30 min exercise performed in each trial (Figure 3). Each session began with a one-minute warm-up (cycling without external workload), followed by a rapid workload increase (~3 s) to the constant-load level corresponding to 50% VO_2_max. Participants cycled continuously for 30 min at a fixed cadence of 70 revolutions per minute.

We evaluated basal blood lactate concentration prior to commencing each trial (baseline) and during exercise. A blood sample was drawn from the right ear lobe during the constant-load exercise at seven and a half minutes and at twenty-two and a half minutes, representing the midpoint of each experimental condition.

### 2.3. Artifact Removal

**Figure 4:**
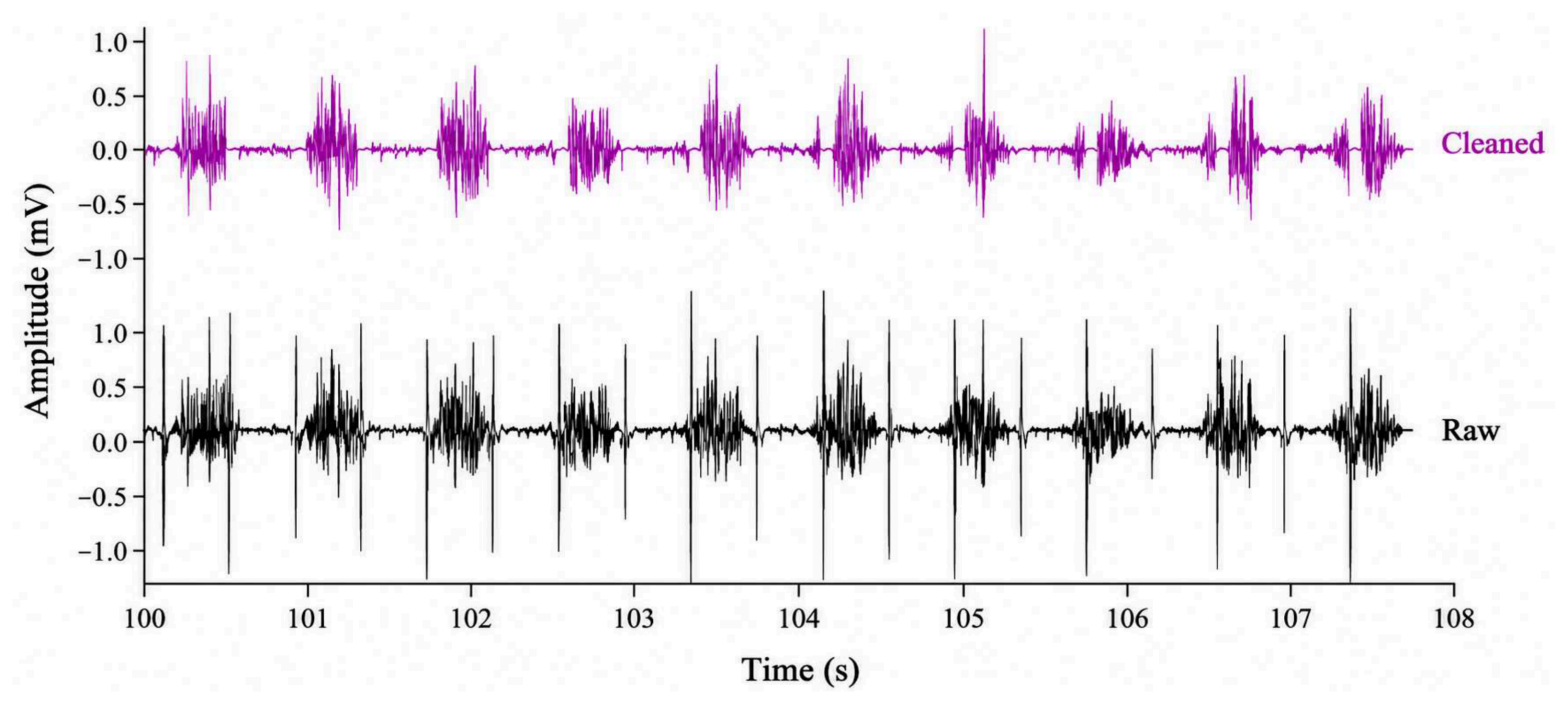
PEMF artifact removal procedure applied to a 10 s raw EMG trace from the RVM. Top panel: Processed signal free from PEMF interference. Bottom panel: Initial recording displaying uncorrected PEMF artifacts prior to filtering.

For an initial filtering of the EMG noise, we used a frequency filter ranging from 20 Hz (high-pass cutoff) to 500 Hz (low-pass cutoff). PEMF artifact removal from raw EMG traces of the RVM and RBF was performed using a four-step algorithm described in Trofè et al. [20]. Specifically, a peak-enhancing procedure, called Smoothed Non-linear Energy Operator (SNEO), was applied to process raw EMG data [22,23], to obtain clean signals (Figure 4). To accentuate transient spikes over the baseline EMG activity, numerical differentiation was applied to the envelope. This operation markedly attenuated the smoother background EMG components relative to the sharp local maxima. Initial peak candidates were detected by applying an absolute amplitude threshold established at 12.5% (1/8) of the global signal peak, combined with a minimal inter-peak interval constraint of 400 ms (corresponding to 80% of the nominal 500 ms stimulation cycle). While this permissive criterion occasionally permitted false positives, it systematically minimized the risk of false negatives. Candidate event timestamps were subsequently refined through an iterative temporal consistency check. The calculated mean stimulation frequency was displayed for preliminary visual confirmation against the nominal 2 Hz baseline. Spurious detections exhibiting inter-peak intervals below 475 ms (representing a tolerance threshold below −5%) were iteratively rejected. Conversely, unassigned stimulation instances were interpolated wherever the elapsed time between consecutive detections fell within the 525 to 975 ms window (approximating 2 × 500 ms within a 25 ms tolerance margin). Following the precise temporal alignment of these artifact coordinates, a blanking window spanning 20 ms prior to and 80 ms following each identified location was applied, setting the corresponding raw EMG segment values directly to zero. Following removal of the PEMF-related interference, muscle activation periods were identified using the onset/offset detection procedure described by Merlo et al. [21]. RMS amplitude was calculated over each detected muscle activation interval, from onset to offset. Therefore, no overlapping RMS calculation windows were used. The complete procedure for artifact removal and RMS computation is implemented in the commercial software EMG Easy Report, version 6.03.8 (Merlo Bioengineering, Parma, Italy) [24,32].

### 2.4. Data Analysis

RMS values of EMG signals were normalized to the individual MVC peak of each muscle for each subject [30,31]. This normalization allowed standardized comparison across participants and conditions. For both muscles (RVM and RBF) and conditions (PEMF ON and PEMF OFF), normalized RMS values were averaged across two temporal phases, that is, the first and the second half of the constant-load exercise, each lasting 15 min. We averaged the values of baseline, warm-up and constant-load exercise for each experimental condition (PEMF ON and PEMF OFF) in both the RBF and RVM.

Statistical analysis was carried out on a final sample of eight participants, as one subject’s data were excluded due to extensive motion artifact contamination. Data were analyzed using IBM SPSS Statistics, version 25.0 (IBM Corp., Armonk, NY, USA). Descriptive statistics were used to summarize study variables. The distribution of continuous variables was tested with the Shapiro–Wilk test. Normally distributed variables are reported as means ± standard deviation (SD). Given the repeated and nested nature of the data, linear mixed-effects models (LMMs) were applied with muscle (RVM and RBF), trial (ON–OFF and OFF–ON) and time (conditions 1, 2, 3, 4) as fixed factors to evaluate temporal effects of PEMF stimulation during the 30 min exercise. The variable “subject” was included as a random intercept to account for repeated measures within individuals. When significant main effects or interactions were detected, pairwise post hoc comparisons were conducted with Bonferroni correction. Statistical significance was set at *p* < 0.05.

Blood lactate values were analyzed by one-way ANOVA and a one-tailed paired *T*-test. For all analyses, effect sizes were expressed as marginal (R^2^m) and conditional (R^2^c) coefficients of determination for LMMs, and as η^2^ or Cohen’s dz for ANOVA and paired *t*-tests [33,34]. Confidence intervals (95% CI) were computed for all effect sizes to provide an estimate of practical significance beyond *p*-values. Statistical significance was set at *p* < 0.05.

## 3. Results

Eight participants completed the four PEMF conditions for a total of 32 recordings (Figure 3): PEMF ON 1st half of exercise (Condition 1), PEMF OFF 2nd half of exercise (Condition 2), PEMF OFF 1st half of exercise (Condition 3), PEMF ON 2nd half on exercise (Condition 4). Data from one subject were excluded from the analysis due to motion artifacts (Figure 1).

**Figure 5:**
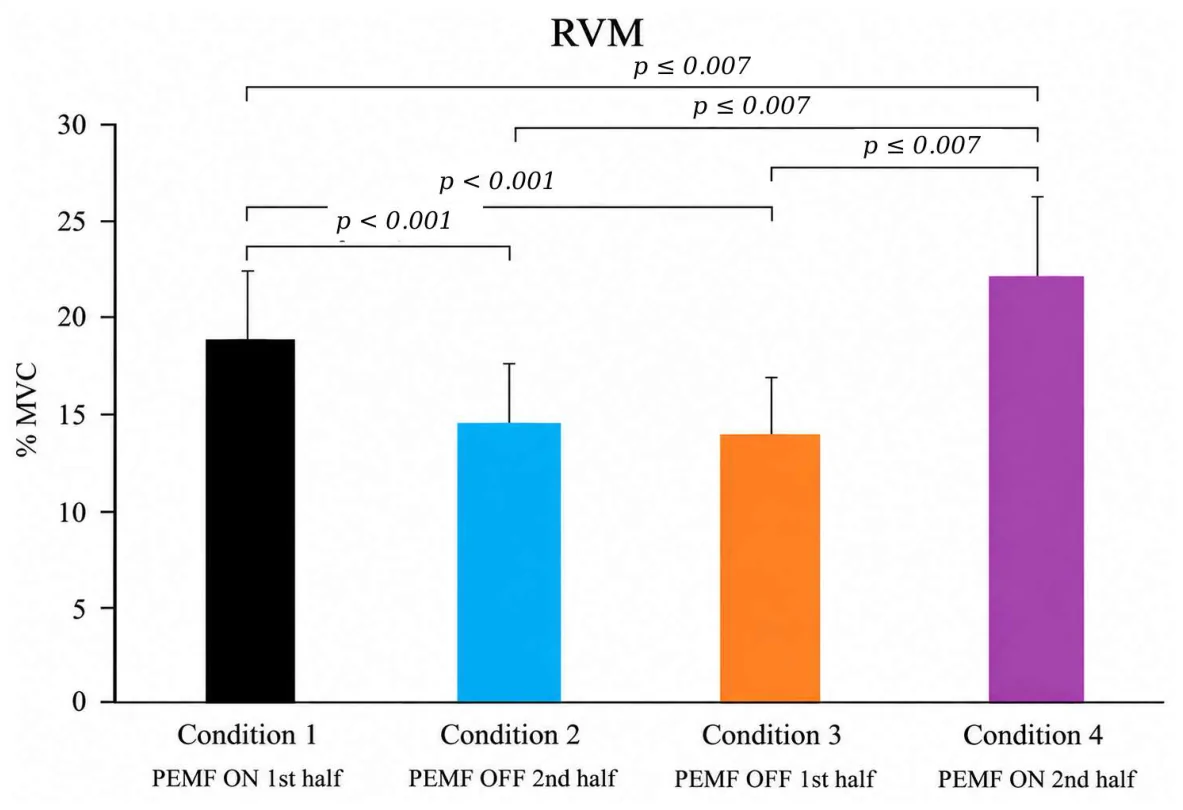
Bar graphs representing the normalized EMG activity (%MVC, root-mean-square values) of the RVM during constant-load exercise, across experimental conditions 1–4 of the 30 min exercise. Data are shown as mean ± SD with significant *p* values. Color codes are the same as in Figure 3.

LMMs showed a significant main effect of PEMFs on EMG amplitude of the RVM (*F*(3,7) = 112.060, *p* < 0.001). The model explained 51% of the variance through fixed effects and 60% overall (R^2^m = 0.51 [95% CI:0.35–0.68], R^2^c = 0.60 [95% CI:0.42–0.73]). The estimated marginal means, expressed as % of MVC ± SD, were: Condition 1 = 18.9 ± 3.8; Condition 2 = 14.3 ± 3.6; Condition 3 = 13.9 ± 3.1; and Condition 4 = 21.8 ± 4.3. Pairwise comparisons (Bonferroni-adjusted) indicated the following: Condition 4 > 1, 2, and 3 (all *p* ≤ 0.007). Condition 1 > 2 and 3 (both *p* < 0.001). Condition 3 vs. Condition 2 was not significant (*p* = 1.000) (Figure 5).

**Figure 6:**
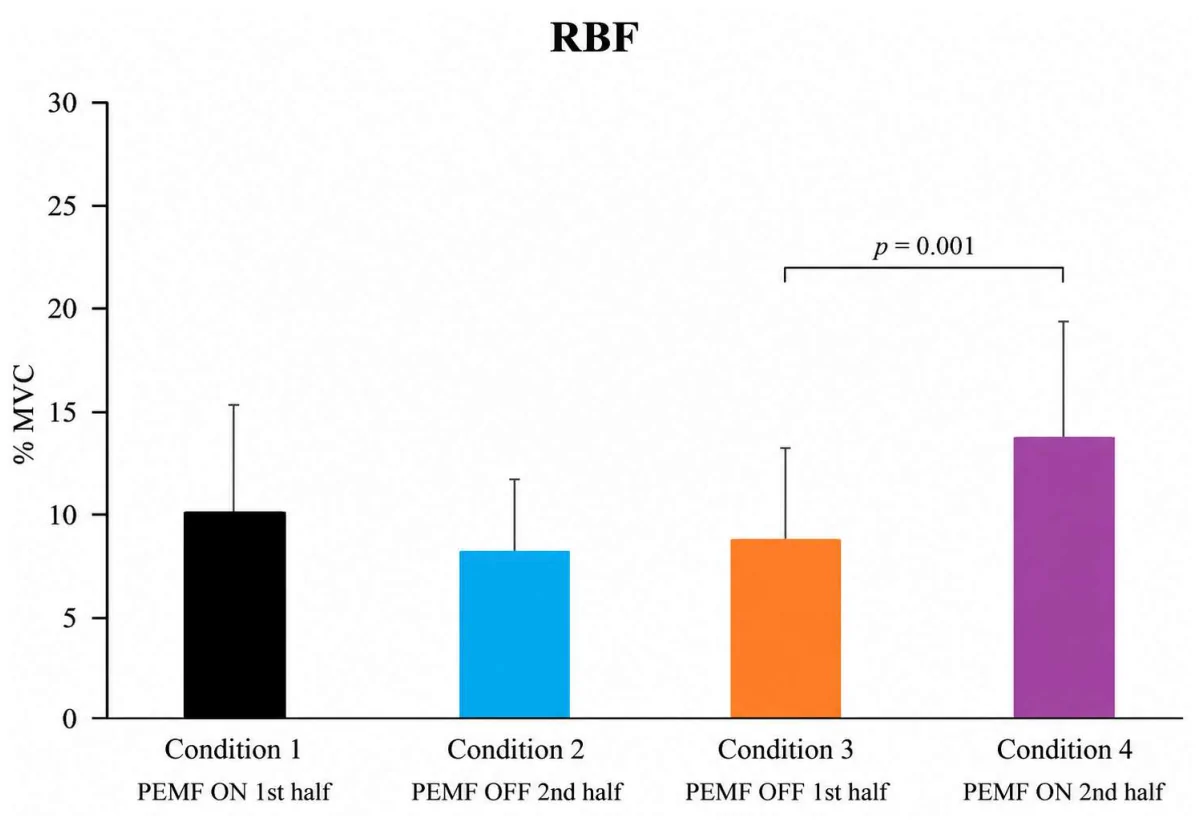
Bar graphs represent the normalized EMG activity (%MVC, root-mean-square values) of the RBF during constant-load exercise, across experimental conditions 1–4 of the 30 min continuous exercise. Data are shown as mean ± SD with significant *p* values. Color codes are the same as in Figure 3.

LMMs showed a significant main effect of PEMFs on EMG amplitude of the RBF (*F*(3,7) = 17.843, *p* = 0.001). The model explained 50% of the variance through fixed effects and 68% overall (R^2^m = 0.50 [95% CI:0.30–0.66], R^2^c = 0.68 [95% CI:0.49–0.80]). The estimated marginal means, expressed as % of MVC ± SD, were: Condition 1 = 9.8 ± 5.3; Condition 2 = 8.2 ± 3.3; Condition 3 = 8.6 ± 4.3; and Condition 4 = 13.4 ± 6.1. Bonferroni-adjusted pairwise comparisons showed that Condition 4 > Condition 3 (*p* = 0.001) (Figure 6).

**Figure 7:**
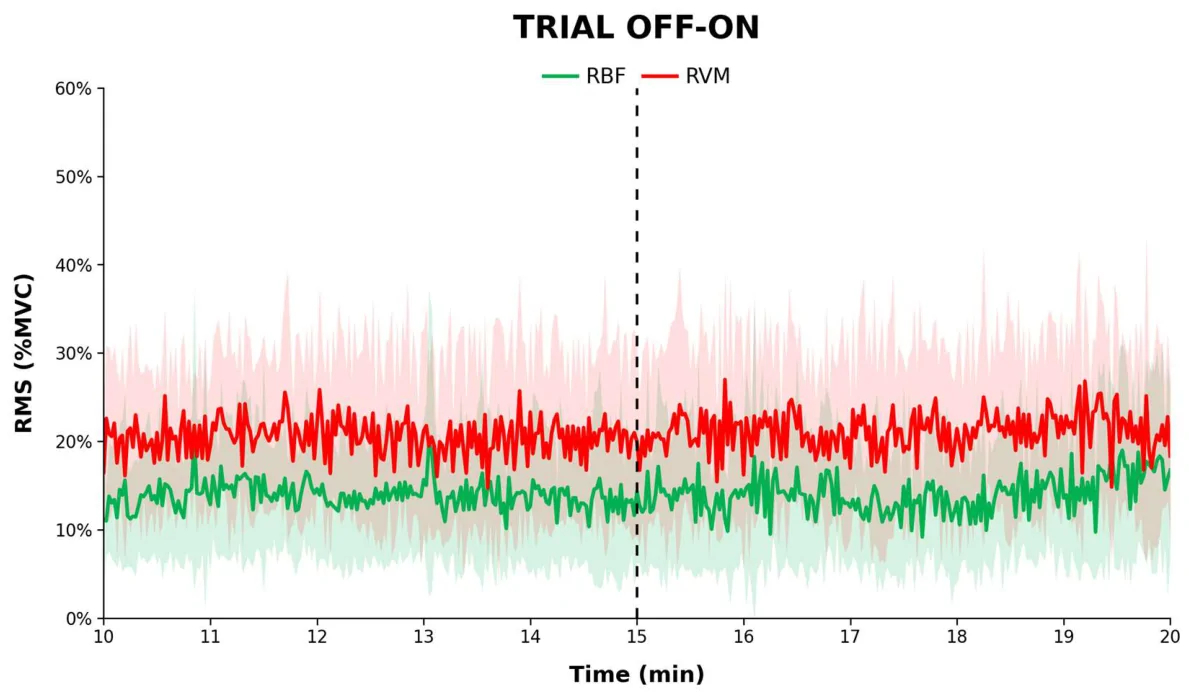
Temporal profile of normalized EMG activity around the PEMF OFF–ON transition. RMS amplitude, expressed as percentage of maximal voluntary contraction (%MVC), is shown for the right vastus medialis (RVM, red) and right biceps femoris (RBF, green) from minute 10 to minute 20 of the 30 min constant-load exercise. The traces represent the averaged conditions in all subjects. The vertical dashed line indicates minute 15, when PEMF stimulation was switched from OFF to ON. Solid lines represent mean values and shaded areas represent the corresponding standard deviation (SD).

**Figure 8:**
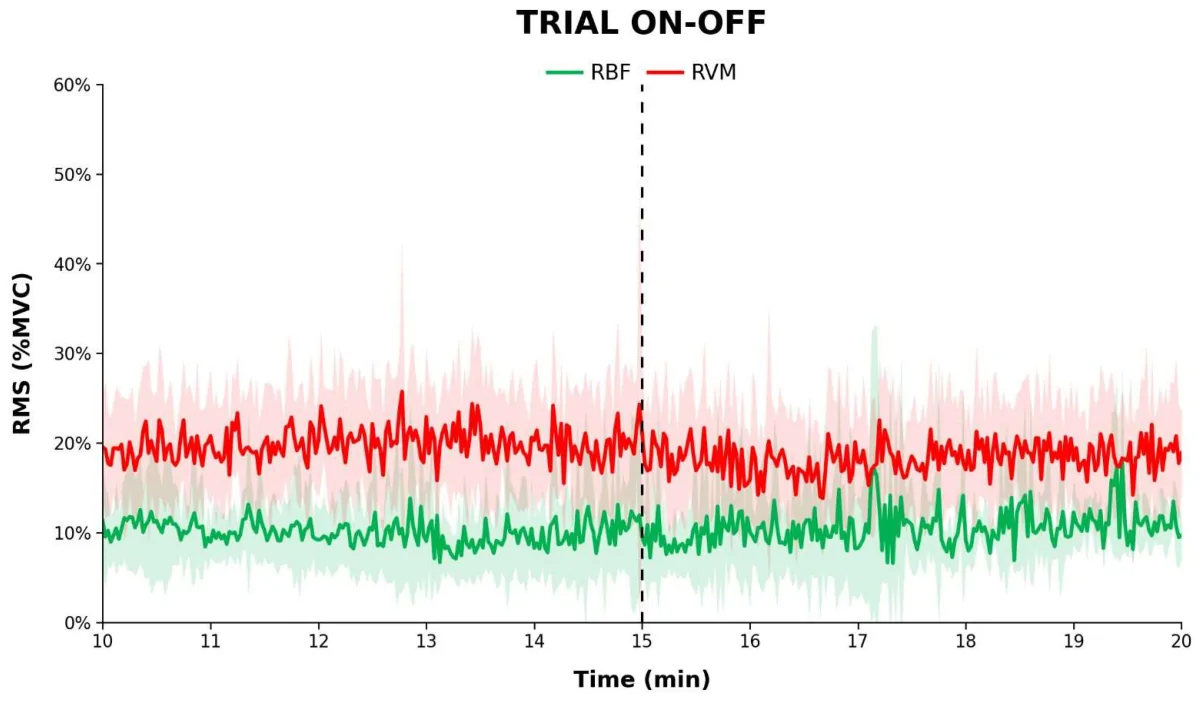
Temporal profile of normalized EMG activity around the PEMF ON–OFF transition. RMS amplitude, expressed as percentage of maximal voluntary contraction (%MVC), is shown for the right vastus medialis (RVM, red) and right biceps femoris (RBF, green) from minute 10 to minute 20 of the 30 min constant-load exercise. The traces represent the averaged conditions in all subjects. The vertical dashed line indicates minute 15, when PEMF stimulation was switched from ON to OFF. Solid lines represent mean values and shaded areas represent the corresponding standard deviation (SD).

To further illustrate the temporal evolution of muscle activation around the change in PEMF stimulation, normalized RMS values were plotted over a 10 min interval encompassing the transition at minute 15 (Figure 7 and Figure 8). A clear change in the temporal pattern of muscle activation was evident around the PEMF transition in both experimental protocols. In the OFF–ON trial, EMG activity tended to increase following PEMF activation, whereas in the ON–OFF trial the opposite pattern was observed following PEMF deactivation. These changes were more evident for the RVM, which also showed consistently greater normalized EMG amplitude than the RBF. The time-resolved traces provide a descriptive visualization of the modulation of muscle activity associated with switching PEMF stimulation.

**Figure 9:**
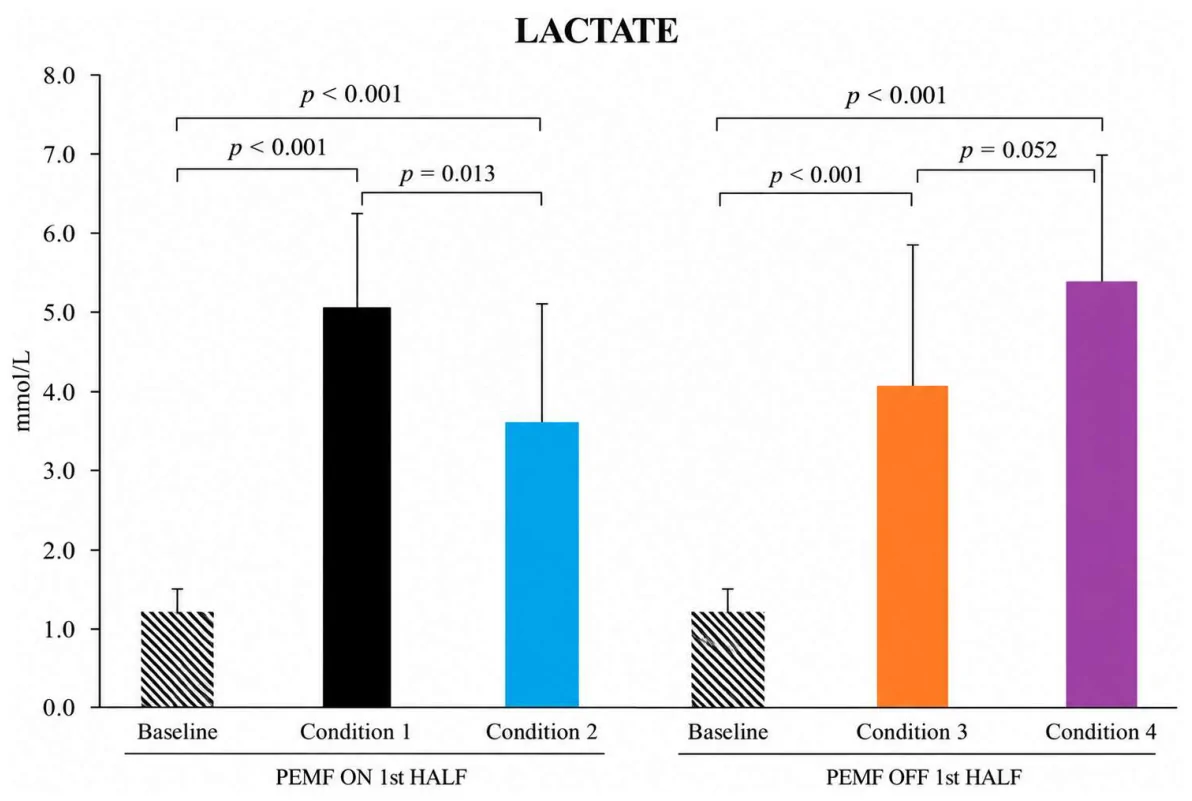
Bar graph representing blood lactate values at baseline and experimental conditions during the constant-load exercise. Data are shown as mean ± SD with significant *p* values. Color codes are the same as in Figure 3.

A one-way analysis of variance of blood lactate concentration showed significant differences across baseline and experimental conditions (F(5,42) = 13.99, *p* < 0.001, η^2^ = 0.63 [95% CI: 0.40–0.75]). Paired one-tailed *t*-tests showed that blood lactate concentrations were significantly higher than baseline in all experimental conditions (Figure 9): Condition 1 vs. baseline, t(7) = 10.43, *p* < 0.001, Cohen’s dz = 3.69 [95% CI: 1.67–5.69]; Condition 2 vs. baseline, t(7) = 5.26, *p* < 0.001, Cohen’s dz = 1.86 [95% CI: 0.66–3.02]; Condition 3 vs. baseline, t(7) = 4.61, *p* = 0.001, Cohen’s dz = 1.63 [95% CI: 0.52–2.69]; and Condition 4 vs. baseline, t(7) = 8.35, *p* < 0.001, Cohen’s dz = 2.95 [95% CI: 1.28–4.60]. Within-trial comparisons showed higher lactate concentrations in Condition 1 than in Condition 2 (t(7) = 2.81, *p* = 0.013, Cohen’s dz = 0.99 [95% CI: 0.11–1.83]). Lactate concentrations were also higher in Condition 4 than in Condition 3, although this difference did not reach statistical significance (t(7) = 1.87, *p* = 0.052, Cohen’s dz = 0.66 [95% CI: −0.13–1.42]).

## 4. Discussion

During moderate-intensity constant-load exercise, active PEMF stimulation (conditions 1 and 4) elicited higher muscle activity compared to the inactive PEMF (conditions 2 and 3) in both RVM and RBF muscles. Moreover, when the PEMF was applied during the latter phase of exercise (Condition 4), its effect was more pronounced. Active PEMF stimulation also resulted in elevated blood lactate concentrations. The observed increase in neuromuscular activation amplitude may reflect enhanced motor unit recruitment. This augmented muscular response could be attributed to PEMF-induced modulation of muscle fiber contraction mechanisms.

### 4.1. Effect of PEMF Timing on Muscle Activity

The results showed that PEMF consistently increased RVM activity, with a greater effect observed when stimulation was applied during the latter 15 min of exercise (Condition 4) compared with the initial phase (Condition 1). This finding indicates that PEMFs exerted a more pronounced influence on muscle activation when delivered after 15 min of cycling. In contrast, significant effects on RBF activity emerged only in Condition 4. In the present study, the moderate-intensity exercise protocol primarily engaged the RVM, while RBF activity remained relatively low.

The functional contribution of the biceps femoris during cycling is complex, as lower-limb muscle activation patterns are influenced by several factors, including cadence, crank position, workload, training status, the shoe–pedal interface, and rider posture [35,36]. In particular, changes in cadence and cycling configuration have been shown to modify the timing and duration of biceps femoris activation [36], while increasing workload alters the activation of both knee extensors and flexors, including the vastus medialis and biceps femoris [37]. As a biarticular muscle, the biceps femoris also contributes to coordination between the hip and knee and to the redistribution of mechanical energy during the pedal cycle [38]. In the present study, the submaximal workload elicited substantially greater activation of the vastus medialis, a primary contributor during the power phase of pedaling, whereas biceps femoris recruitment remained comparatively lower. Nevertheless, the biceps femoris was retained in the analysis because its distinct functional role during cycling provides complementary information on muscle-specific responses to PEMF stimulation.

For both muscles, the increased EMG amplitude observed under PEMF stimulation may reflect modulation of neuromuscular excitability and muscle recruitment. Experimental studies in skeletal muscle cells have shown that time-varying magnetic fields can induce transient changes in membrane potential, promote Na^+^ influx through voltage-gated sodium channels, and increase cytosolic Ca^2+^ levels [10,39,40]. Other studies [41] using PEMF exposure have identified downstream calcium-dependent and mitochondrial signaling as potential mechanisms influencing skeletal muscle function. These mechanisms were not directly investigated in the present study and therefore remain speculative in the context of our findings. The current results should be interpreted as evidence of acute modulation of muscle activation.

### 4.2. PEMF Effects on Muscle Activation and Systemic Metabolic Responses

In agreement with the present results, previous studies have reported increased systemic blood lactate concentrations during exercise performed under PEMF stimulation [17,19]. In this study, lactate was measured from the ear lobe and therefore represents a systemic measurement. Consequently, the observed increase in blood lactate cannot be directly attributed to the stimulated muscles or to specific muscle fiber types. The higher systemic lactate concentrations observed during PEMF stimulation may reflect an altered glycolytic contribution during exercise or, alternatively, may simply result from the greater muscle activation indicated by the increased EMG amplitude and the consequent increase in lactate production. The present data do not allow us to distinguish between these possible explanations or to determine whether PEMF specifically affected glycolysis in particular muscle fiber types or intramuscular acid–base balance. Direct local metabolic measurements would be required to clarify these mechanisms. Nevertheless, the observation of concurrent changes in muscle activation and systemic lactate may be particularly relevant in sedentary individuals, who typically exhibit reduced muscle efficiency and exercise capacity. In support of a possible metabolic contribution, experimental studies in animal models have shown that PEMF can modulate cellular energy metabolism through pathways involving NAD signaling and ATP synthesis [42]. Although these findings cannot be directly extrapolated to the present human data, they provide a plausible biological framework for future investigations of the metabolic effects of PEMF during exercise.

From a practical perspective, the present findings may be particularly relevant for sedentary individuals, who commonly exhibit low cardiorespiratory fitness, muscular strength, and exercise capacity [7]. In this population, PEMF could represent a complementary strategy to enhance neuromuscular activation during moderate-intensity exercise without requiring an increase in the external workload. This may be of interest during the initial phases of exercise programs, when maintaining a tolerable exercise intensity while providing an adequate muscular stimulus can be challenging. Our findings, together with previous evidence showing PEMF-related increases in muscle activation in sedentary individuals [20], suggest that PEMF may act as an adjunct to exercise. Whether the acute increase in muscle activation observed here translates into greater training adaptations, improved functional capacity, or long-term health benefits remains to be established in longitudinal studies.

### 4.3. Limitations

The small sample size (*n* = 8) represents the most important limitation of this study, restricting the generalizability of the findings. Therefore, effect sizes were reported using marginal and conditional R^2^ values to provide more reliable estimates of explained variance in mixed-effects models than traditional η^2^ metrics. The obtained R^2^m (0.48–0.62) and R^2^c (0.55–0.68) indicate large effects of PEMF stimulation on EMG activity. These values suggest that a substantial proportion of the variability in muscle activation was explained by the PEMF condition, whereas the remaining variance was primarily attributable to inter-individual differences. The use of R^2^ metrics also mitigates the potential inflation of effect size estimates commonly associated with small-sample, within-subject experimental designs.

An additional limitation is that the study included only young sedentary males. This choice limits the generalizability of the present findings to females. Future studies should specifically investigate young sedentary women to determine whether PEMF-induced changes in muscle activity differ between sexes and whether these responses are influenced by menstrual-cycle-related hormonal fluctuations.

## 5. Conclusions

The findings of this study support the initial hypothesis that PEMF stimulation exerts differential effects on muscle activation depending on the timing of its application. These results align with previous evidence demonstrating a strong modulatory influence of PEMF on muscle function during physical exertion. Importantly, the present study emphasizes that the timing of stimulation is a critical determinant of the magnitude of the PEMF effect.

Beyond contributing to a better understanding of the physiological responses to PEMF during exercise, these findings may have practical implications for sedentary individuals initiating exercise programs, as well as for physically active individuals and professionals involved in exercise prescription.

## Figures and Tables

**Table 1:** Descriptive characteristics of study participants. Data are presented for each subject, including age, maximal oxygen consumption (VO_2_max, expressed in mL/min/kg), workload at exhaustion (watts), body weight (kg), height (cm), and body mass index (BMI). Mean values, standard deviations (SD), and standard errors of the mean (SEM) are also reported.

Subjects	Age (years)	VO_2_max (mL/min/kg)	Workload (watt)	Weight (kg)	Height (cm)	BMI
L.B	32	33.9	108	76	175	24.8
E.M	30	31.9	113	72	180	22.2
A.T	34	33.2	100	77	177	24.6
S.B	34	33.3	108	87	178	27.5
M.S	23	34.4	88	62	170	21.5
A.A	20	33.9	85	67.5	173	22.6
S.P	25	39.5	105	64	164	23.8
E.B	35	30.5	83	79	173	26.4
S.I	32	35.3	106	74	168	26.2
Mean	29.4	34.0	99.6	73.2	173.1	24.4
SD	5.4	2.5	11.3	7.8	5.1	2.1
SEM	1.8	0.8	3.8	2.6	1.7	0.7

## Data Availability

The data that support the findings of this study are not openly available due to their sensitive nature and are available from the corresponding author upon reasonable request. Data are located in controlled-access data storage at the University of Bologna (Italy).
